# BM-1197: A Novel and Specific Bcl-2/Bcl-xL Inhibitor Inducing Complete and Long-Lasting Tumor Regression *In Vivo*

**DOI:** 10.1371/journal.pone.0099404

**Published:** 2014-06-05

**Authors:** Longchuan Bai, Jianfang Chen, Donna McEachern, Liu Liu, Haibin Zhou, Angelo Aguilar, Shaomeng Wang

**Affiliations:** 1 University of Michigan Comprehensive Cancer Center and Department of Internal Medicine, University of Michigan, Ann Arbor, Michigan, United States of America; 2 University of Michigan Comprehensive Cancer Center and Department of Pharmacology, University of Michigan, Ann Arbor, Michigan, United States of America; 3 University of Michigan Comprehensive Cancer Center and Department of Medicinal Chemistry, University of Michigan, Ann Arbor, Michigan, United States of America; University of Nebraska Medical Center, United States of America

## Abstract

Bcl-2 and Bcl-xL are critical regulators of apoptosis that are overexpressed in a variety of human cancers and pharmacological inhibition of Bcl-2 and Bcl-xL represents a promising strategy for cancer treatment. Using a structure-based design approach, we have designed BM-1197 as a potent and efficacious dual inhibitor of Bcl-2 and Bcl-xL. BM-1197 binds to Bcl-2 and Bcl-xL proteins with K_i_ values less than 1 nM and shows >1,000-fold selectivity over Mcl-1. Mechanistic studies performed in the Mcl-1 knockout mouse embryonic fibroblast (MEF) cells revealed that BM-1197 potently disassociates the heterodimeric interactions between anti-apoptotic and pro-apoptotic Bcl-2 family proteins, concomitant with conformational changes in Bax protein, loss of mitochondrial membrane potential and subsequent cytochrome c release to the cytosol, leading to activation of the caspase cascade and apoptosis. BM-1197 exerts potent growth-inhibitory activity in 7 of 12 small cell lung cancer cell lines tested and induces mechanism-based apoptotic cell death. When intravenously administered at daily or weekly in H146 and H1963 small-cell lung cancer xenograft models, it achieves complete and long-term tumor regression. Consistent with its targeting of Bcl-xL, BM-1197 causes transit platelet reduction in mice. Collectively, our data indicate that BM-1197 is a promising dual Bcl-2/Bcl-xL inhibitor which warrants further investigation as a new anticancer drug.

## Introduction

Impaired apoptosis is one of the hallmarks of cancer and contributes to tumor progression and resistance to conventional cancer therapy [1]. One of the main apoptosis pathways is the mitochondria-mediated intrinsic pathway, which is defined by mitochondrial outer membrane permeabilization (MOMP). On the molecular level, MOMP is controlled by the dynamic interactions between a set of pro-apoptotic and anti-apoptotic B-cell lymphoma-2 (Bcl-2) proteins. Proteins of the anti-apoptotic Bcl-2 family, including Bcl-2, Bcl-xL, Bcl-w, Mcl-1, and Bfl1/A1, inhibit MOMP by sequestering pro-apoptotic Bcl-2 family members, such as Bax, Bak, Bim, Bid, and Puma [2]. Thus, upregulation of anti-apoptotic Bcl-2 proteins and/or down-regulation of pro-apoptotic proteins can confer resistance to apoptotic stimuli on tumor cells [3], [4]. Indeed, one or more of these anti-apoptotic Bcl-2 proteins is overexpressed in human cancers, resulting in resistance to chemotherapy and radiation [4], [5], [6], [7], [8], [9], [10]. Therefore, pharmacological inhibition of one or more of these anti-apoptotic Bcl-2 family proteins has been pursued as a novel cancer therapeutic strategy with the goal of overcoming apoptosis resistance of tumor cells.

Non-peptide, small-molecule inhibitors have been developed which target one or more of these anti-apoptotic Bcl-2 proteins through disruption of the protein-protein interactions between anti- and pro-apoptotic Bcl-2 proteins [11], [12], [13], [14], [15], [16], [17], [18]. ABT-737 [11] and its orally active analog, ABT-263 (navitoclax) [13] are arguably two of the most effective dual Bcl-2 and Bcl-xL inhibitors. ABT-737 and ABT-263 bind to Bcl-2 and Bcl-xL and show high selectivity over Mcl-1 and A1. On the other hand, ABT-199 selectively targets Bcl-2 over Bcl-xL and other anti-apoptotic Bcl-2 members [18], while WEHI-539 [14] and BXI-72 [19] demonstrate high potency and specificity for Bcl-xL. Some selective Mcl-1 inhibitors have also been recently reported [20]. Among highly potent and specific small-molecule inhibitors targeting these anti-apoptotic Bcl-2 proteins, ABT-263 [13], [21] and ABT-199 [18] have been advanced into clinical development and both compounds have demonstrated impressive antitumor activity as single agents in patients with chronic lymphocytic leukemia, in which the cells are primarily dependent upon Bcl-2 for survival. These encouraging clinical data for ABT-263 and ABT-199 provide strong evidence that pharmacological targeting of critical anti-apoptotic Bcl-2 proteins has promise for the treatment of human cancers.

To date, the only potent and specific dual Bcl-2/Bcl-xL inhibitor that has been advanced into clinical development is ABT-263 [13], [21]. Although this compound binds to both recombinant Bcl-2 and Bcl-xL with K_i_ values, determined in biochemical assays of <1 nM, recent data suggest that more potent and efficacious dual small-molecule inhibitors of Bcl-2 and Bcl-xL may be needed in order to successfully target tumor cells whose survival is protected by Bcl-xL alone or by both Bcl-2 and Bcl-xL. First, due to its strong binding to albumin, approximately 100-fold higher concentrations of ABT-263 are required for it to induce apoptosis in whole blood rather than in standard cell culture conditions [22]. Second, while ABT-263 is effective in antagonizing Bcl-2, it is relatively less effective in antagonizing Bcl-xL [23]. Therefore, development of new, dual Bcl-2 and Bcl-xL inhibitors with improved potency and efficacy will provide an opportunity to effectively target tumor cells whose survival is protected by both Bcl-2 and Bcl-xL or by Bcl-xL alone.

Employing a structure-based design approach, we have designed a class of potent, small-molecule dual inhibitors of Bcl-2 and Bcl-xL. For example, BM-1074 was shown to bind to Bcl-2 and Bcl-xL with K_i_ values of <1 nM [16]. BM-1074 inhibits cancer cell growth with IC_50_ values of 1–2 nM in four small-cell lung cancer cell lines sensitive to ABT-263 and is >10-times more potent than ABT-263. Significantly, BM-1074 is capable of achieving rapid, complete, and durable tumor regression in the H146 small-cell lung cancer xenograft model in severe combined immunodeficiency (SCID) mice. These data suggest that BM-1074 represents a class of promising dual Bcl-2 and Bcl-xL inhibitors. Toward developing a highly potent and efficacious dual Bcl-2 and Bcl-xL inhibitor for cancer treatment, we have further optimized BM-1074 for its solubility and pharmacokinetic properties and this has led to BM-1197. We report herein our detailed investigation of BM-1197 in which we studied its mechanism of action and therapeutic potential as a single agent *in vitro* and *in vivo*.

## Materials and Methods

### Ethics Statement

All the in vivo studies were performed under an animal protocol 9094 approved by the University Committee on Use and Care of Animals of the University of Michigan, in accordance with the recommendations in the Guide for the Care and Use of Laboratory Animals of the National Institutes of Health.

### Reagents and Antibodies

ABT-263 was obtained from Selleck Chemicals (Houston, TX) and JC-1 from Cayman Chemicals (Ann Arbor, MI). Rabbit anti-Bcl-xL (#2764), Bcl-2 (#2870), Bim (#2819), PARP (#9532) and caspase-3 (#9665), and mouse anti-caspase-9 (#9808) were from the Cell Signaling Technology (Danvers, MA). Rabbit anti-Bak (sc-832), Bim (sc-11425), Puma (sc-28226) and Bcl-2 (sc-492), mouse anti-Puma (sc-374223), Bax (sc-70405, sc-23959), Bcl-2 (sc-509), Bcl-xL 9sc-8392), cytochrome c (sc-13560), and β-actin (sc-1616), and goat anti-Puma (sc-19187) were from Santa Cruz Biotechnology (Santa Cruz, CA). Rabbit anti-Bim (#559685), mouse anti-cytochrome c (#556433), and hamster anti-mouse Bcl-2 (#554218) were provided by BD Pharmingen. Rat anti-Bim (#804-528) was from Enzo Life Sciences (Farmingdale, NY) and Mouse ant-Bak (#06-536) was from EMD Millipore (Billerica, MA).

### Cell Culture, Cell Growth Inhibition, Mitochondrial Membrane Potential and Apoptosis Assays

All the SCLC cell lines were purchased from American Type Culture Collection (Manassas, VA) and cultured as recommended. All these cell lines were passaged fewer than 6 months after purchase. MCL1 knockout (*MCL1^−/−^*) MEF cells were kindly provided by Dr. David Huang (The Walter and Eliza Hall Institute of Medical Research, Melbourne, Australia). Cell growth inhibition was evaluated by means of a WST-8 assay (Dojindo, Rockville, MD) as described previously [24]. Mitochondrial membrane potential was determined by JC-1 staining using a JC-1 Mitochondrial Membrane Potential Assay kit (Cayman). Apoptosis analysis was performed using an Annexin V/propidium iodide (PI) apoptosis detection kit (Roche Applied Sciences, Indianapolis, IN).

### RNA Interference, Immunoblotting and Immunoprecipitation

ON-TARGETplus SMARTpools for mouse Bax and Bak and non-targeting negative control siRNAs were obtained from Dhamarcon. Transfection was performed using Lipofectamine RNAiMAX (Invitrogen). Lentiviral vector for scrambled and human MCL1 shRNA and lentivirus infection were described previously [25]. Immunoblotting was performed using a published procedure [26]. For immunoprecipitation assays, cells lysed with CHAPS buffer (150 mM NaCl, 10 mM HEPES [pH 7.4], 0.5% Chaps) or Triton buffer (150 mM NaCl, 10 mM HEPES [pH 7.4], 0.5% Triton X-100) supplemented with Complete protease inhibitors (Roche). Immunoprecipitation and immunoblot assays were performed with the indicated antibodies. Active Bax was detected by immunoprecipitation with the Bax 6A7 antibody, which recognizes only the conformationally changed Bax protein [27].

### Cytochrome c Release Assay

Cells were treated with drugs to the indicated time points, washed with PBS, and re-suspended in digitonin buffer (20 mmol/L HEPES [pH 7.4], 75 mM NaCl, 1.5 mM MgCl_2_, 1 mM EDTA, 350 µg/ml digitonin, and 250 mM sucrose) supplemented with protease inhibitors. Cytosolic fractions were separated from organelle membrane fraction by centrifugation at 13,000 rpm for 10 min. The cytosolic fractions were analyzed by immunoblotting.

### Cellular Thermal Shift Assay (CETSA)

Drug-target engagement was assessed using CETSA as described by Molina et al. [28]. Briefly, cells were treated with drugs for 1 h, then harvested, washed with PBS, and resuspended in PBS. The resuspended cells were freeze-thawed 4 times. The soluble fraction (lysate) was separated from cell debris by centrifugation at 20000×g for 20 minutes at 4°C and then analyzed by Western blots.

### *In vivo* Xenograft Studies

SCID mice bearing xenograft tumors were treated with BM-1197 and ABT-263 at the indicated dosing schedules. Tumor sizes were measured 2–3 times per week. Tumor growth inhibition was calculated by using the formula: 100% (mean volume of controls - mean volume of treated)/mean volume of controls.

### Platelet Count in Mice

BM-1197 was administered by IV bolus injected into the tail vein of the mice. Mice were euthanized at different time points with CO_2_, then ∼0.2 ml of blood was drawn by cardiac puncture using an EDTA treated syringe, transferred to an EDTA treated tube and sent immediately for a Complete Blood Count. The samples were read at the University of Michigan Animal Diagnostic Laboratory on a HEMAVET 950FS Hematology Analyzer.

### Statistical Analyses

For the cell growth inhibition assay, data were plotted as mean ± SD, and sigmoid fitted (variable slope). Differences in mean values of cell growth inhibition among different groups were analyzed by two-way analysis of variance. For *in vivo* studies, significance (*P*) was calculated by Student's *t* test. All statistical tests were two-sided, and all statistical analyses were performed using GraphPad Prism 5. The *P* values less than 0.05 are considered statistically significant.

## Results

### BM-1197 is a potent and selective inhibitor of Bcl-2 and Bcl-xL

**Figure 1:**
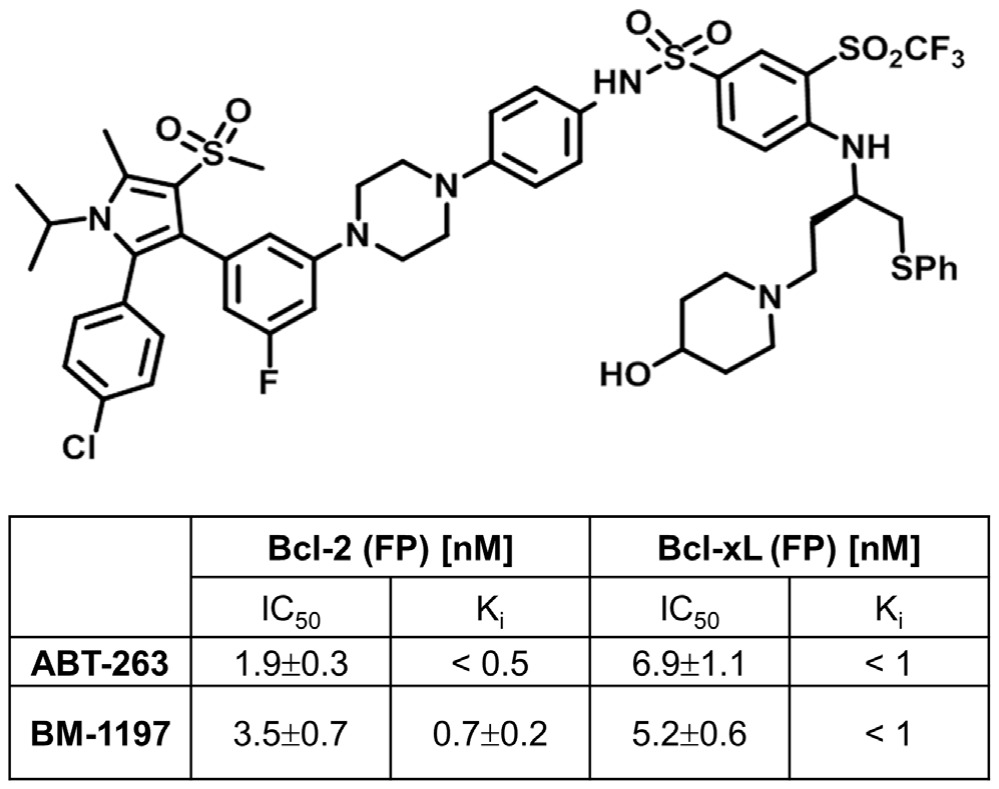
Chemical structure and binding affinities of BM-1197. The binding affinities of BM-1197 and ABT-263 to Bcl-2 and Bcl-xL determined by fluorescence-polarization binding assay. Data are expressed as means ± SD (n≥3).

BM-1197 (**Fig 1**), was synthesized in a similar manner as described for BM-1074 [16]. We evaluated the binding affinities of BM-1197 to Bcl-2, Bcl-xL and Mcl-1 proteins using our optimized fluorescence-polarization (FP) binding assays [16]. BM-1197 binds to Bcl-2 and Bcl-xL with K_i_ <1 nM but fails to bind to Mcl-1 at 2 µM. Hence, BM-1197 has a binding profile, similar to ABT-263 and ABT-737. Of note, the binding affinities of these compounds to Bcl-2 and Bcl-xL proteins are close to the lower limits of the assay and their precise K_i_ values could not be determined accurately using our competitive, FP binding assays [16].

### BM-1197 induces mechanism-based apoptosis in Mcl1-deficient mouse embryonic fibroblast (MEF) cells

**Figure 2:**
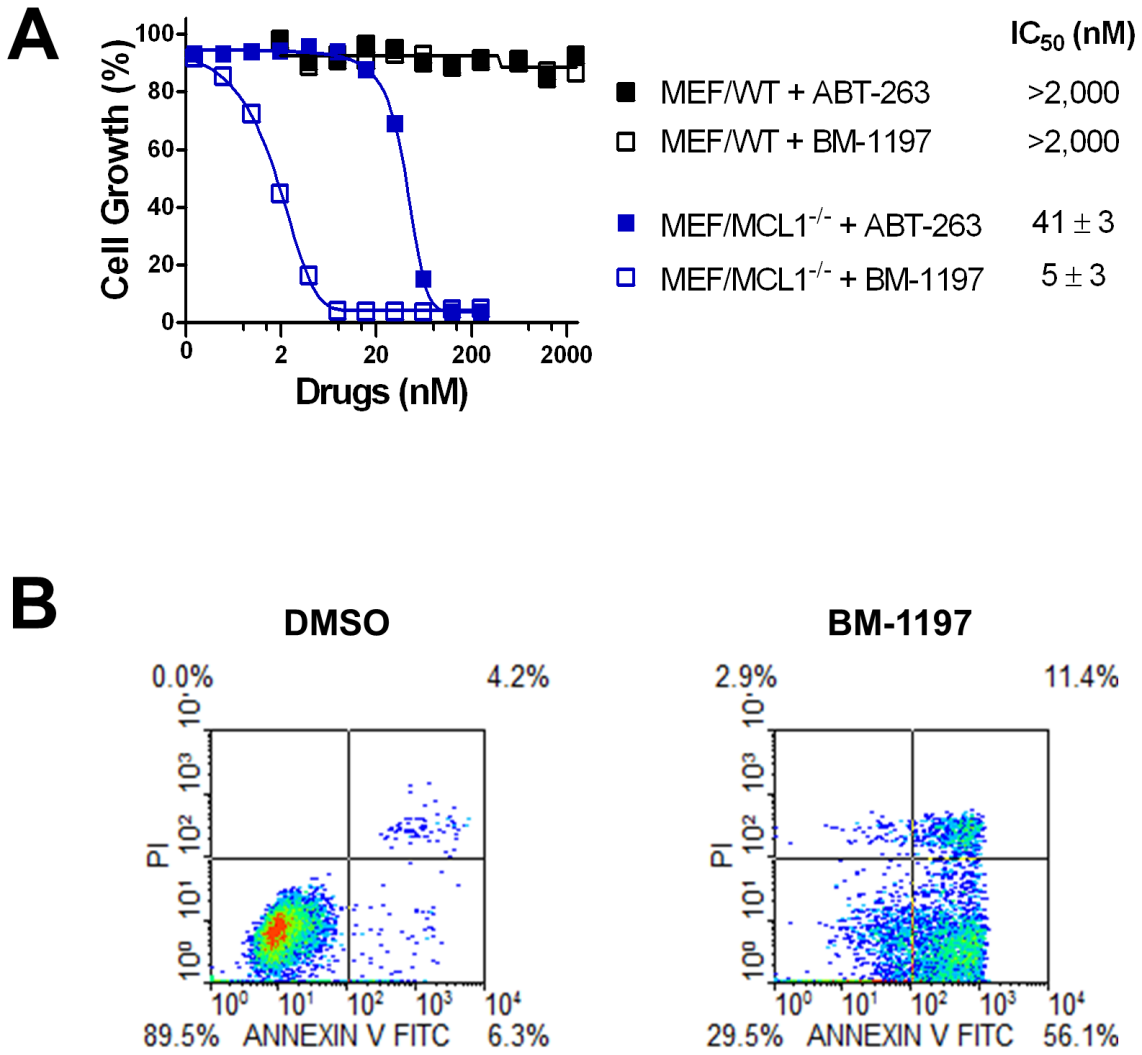
BM-1197 induces apoptosis in MEF/*MCL1* ^−/−^ cells. (A) *MCL1*-wild-type (WT) or –deficient (*MCL1^−/−^*) MEF cells were treated with BM-1197 or ABT-263 for 3 days and growth-inhibitory activity of BM-1197 was evaluated by WST assay. Data are representative of at least three independent experiments. (B) *MCL1^−/−^* cells were treated with 100 nM of BM-1197 for 16 h and stained with Annexin V/PI for flow cytometry.

Wild-type MEF cells and Mcl-1-deficient MEF (MEF/*MCL1^−/−^*) cells have been employed as models to establish the potency and on-target activity for potent and selective Bcl-2 and/or Bcl-xL inhibitors, such as ABT-737, ABT-263 and WEHI-539 [14], [29], [30]. Consistent with our biochemical data that BM-1197 potently binds to Bcl-2 and Bcl-xL but not to Mcl-1, BM-1197 had marginal cytotoxicity against wild-type MEF cells but exerted potent growth-inhibitory activity in the *MCL1^−/−^* cells (**Fig 2A**). In direct comparison, BM-1197 is ∼8 times more potent than ABT-263 in the inhibition of cell growth of *MCL1^−/−^* cells (**Fig 2A**). Annexin V staining revealed that BM-1197 potently induced apoptosis in *MCL1^−/−^* cells (**Fig 2B**).

**Figure 3:**
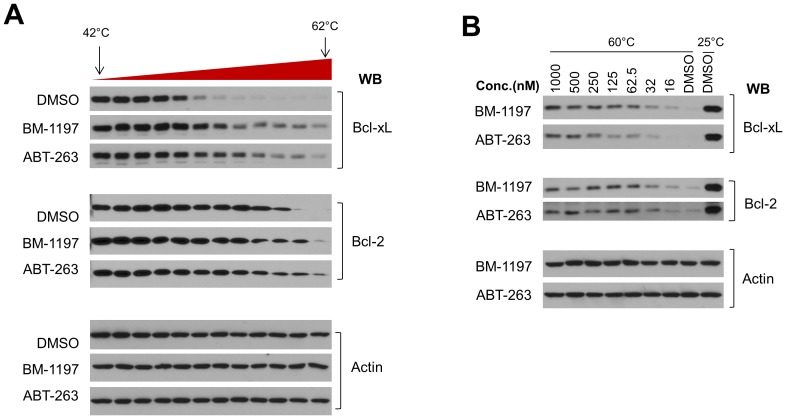
(A) MEF MCL1*^−^* ^/*−*^ cells were treated with DMSO, or 1 µM of ABt-263 or BM-1197 for 1 h. Drug-treated cells were heated at gradient temperature from 42°C to 62°C for 3 min then freeze-thawed 4 times. The soluble fractions of the lysed cells were analyzed by immunoblotting. (B) MEF MCL1*^−^* ^/*−*^ cells were treated with drugs at the indicated doses for 1 h before collected for CETSA.

To validate the drug-target interaction in the cellular context, we performed a cellular thermal shift assay (CETSA) [28]. In the absence of drugs and when subjected to heat denaturing, cellular Bcl-xL and Bcl-2 proteins lost their higher-order structure, unfold and denature at around 52°C and 60°C respectively (**Fig 3A**). In the presence of ABT-263 or BM-1197 at 1 µM, the stabilities of Bcl-xL and Bcl-2 proteins, but not of the control protein Actin, were enhanced (**Fig 3A**). To further determine the cellular potency of ABT-263 and BM-1197 in target engagement, we performed CETSA with different concentrations of these drugs. As shown in **Fig 3B**, BM-1197 efficiently stabilized BCL-xL protein at concentrations as low as 16 nM, but higher concentrations of ABT-263 were required to achieve a similar effect to that of BM-1197. Both drugs effectively increased the thermal stability of Bcl-2 with no significant difference in their potencies (**Fig. 3B**). These data suggest that in cells, BM-1197 is more efficient than ABT-263 in targeting the BCL-xL protein and these two drugs have similar potencies in targeting Bcl-2 protein.

**Figure 4:**
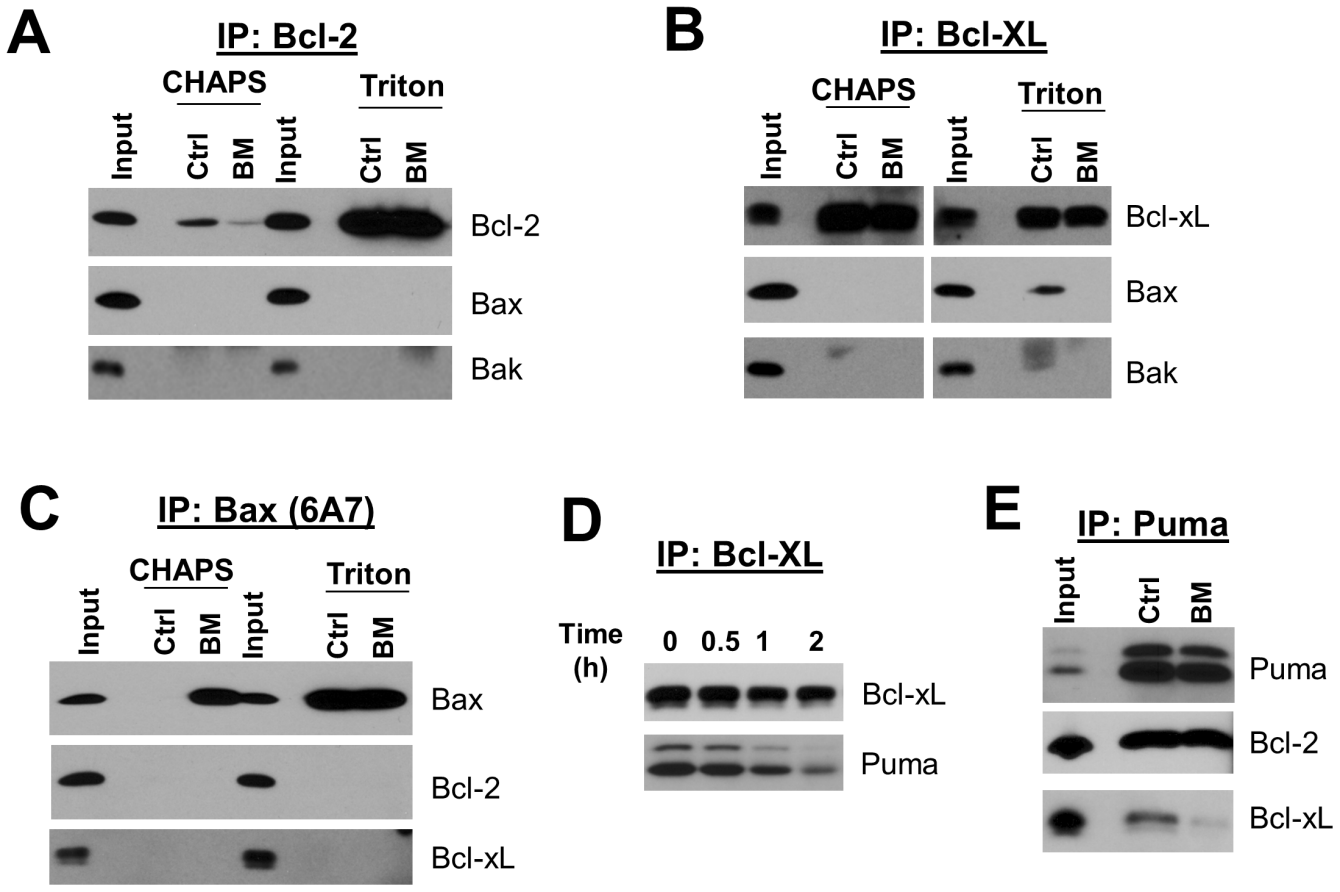
BM-1197 disrupts the association between BCL-XL and PUMA in MEF/*MCL1^−^* ^/−^ cells. (A–C) MCL1*^−^* ^/*−*^ MEF cells were treated with DMSO (ctrl, control) or BM-1197 (BM, 50 nM) for 1 h and CHAPS- or Triton X-100-lysed whole cell extracts were immunoprecipitated with the indicated antibodies. The immunoprecipitates were analyzed by immunoblotting. (D & E) CHAPS lysed whole cell extracts from BM-1197 (50 nM)-treated *MCL1^−/−^* cells were immunoprecipitated with the indicated antibodies, and immunoprecipitates were analyzed for the presence of Bcl-xL and PUMA by immunoblotting.

To further investigate the cellular mechanism of action of BM-1197, co-immunoprecipitation studies of Bcl-xL or Bcl-2 with pro-apoptotic Bcl-2 family proteins were carried out on lysates from BM-1197- or DMSO-treated *MCL1^−/−^* cells. Our studies revealed that Bcl-xL or Bcl-2 failed to associate with either Bax or Bak when the cells were lysed with zwitterionic detergent CHAPS (**Fig 4A–C**), suggesting that these proteins do not heterodimerize in viable *MCL1^−/−^* cells. Instead, Puma was co-immunoprecipitated with Bcl-xL, and this association was diminished in a time-dependent manner by BM-1197 (**Fig 4D**). Reciprocal immunoprecipitation with Puma antibody confirmed that BM-1197 dissociated the interactions between Bcl-xL and Puma (**Fig 4E**).

**Figure 5:**
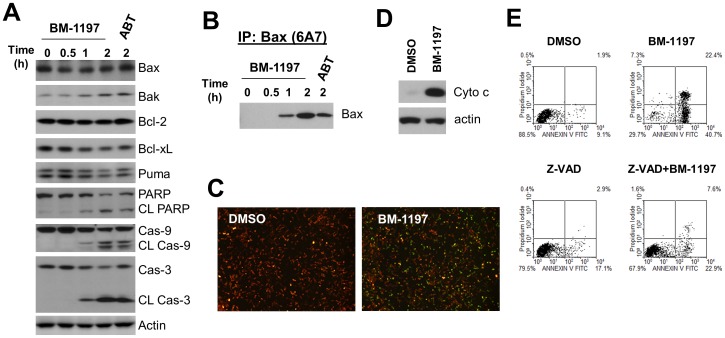
BM-1197 induces BAX translocation and cytochrome c release in MEF/*MCL1* ^−/−^ cells. (A) Cells were treated with BM-1197 (50 nM) or ABT-263 (50 nM) for the indicated time points and whole cell extracts were analyzed by immunoblotting. (B) CHAPS lysed whole cell extracts from BM-1197 (50 nM)-treated *MCL1^−/−^* cells were immunoprecipitated with conformation-sensitive Bax specific antibody 6A7, and immunoprecipitates were analyzed for Bax by immunoblotting. (C) *MCL1^−/−^* cells were treated with BM-1197 (50 nM) for 30 min, then co-incubated with JC-1 (2 µg/ml) for an additional 30 min. Representative images are shown. (D) *MCL1^−/−^* cells were treated with BM-1197 (50 nM) for 1 h and cytosolic fraction was isolated for immunoblotting. (E) MCL1*^−^* ^/*−*^ cells were pretreated with DMSO or 40 µM of Z-VAD for 2 h, then treated with 100 nM of BM-1197 for 16 h and stained with Annexin V/PI for flow cytometry analysis.

Immunoblotting of total cell lysates indicated that overall expression levels of pro- and anti-apoptotic Bcl-2 family proteins were comparable prior to and after BM-1197 treatment (**Fig 5A**). Another pro-apoptotic Bcl-2 member, Bim, which has a role similar to that of Puma, was below the detection limit of the present immunoprecipitation and immunoblotting assays.

One key mechanism for regulation of apoptosis by the Bcl-2 family proteins is to control MOMP (Δψm) and release of cytochrome c through modulation of the pore formation of Bax or Bak protein in the outer mitochondrial membrane (3). Immunoprecipitation of Bax protein with the conformation-sensitive antibody 6A7 demonstrated that treatment with BM-1197 resulted in conformational change in the Bax protein, which occurred as early as 1 h after drug exposure (**Fig 5B**). Further, JC-1 staining revealed a significant increase in the green signal (a surrogate marker of loss of mitochondrial Δψm) in BM-1197-treated cells when compared with control cells, indicating the loss of MOMP in these cells as a result of BM-1197 treatment (**Fig 5C**). Immunoblotting analysis of the cellular cytosol fraction demonstrated an accumulation of cytochrome c in the cytosol fraction, indicating the release of cytochrome c from mitochondria to cytosol upon BM-1197 treatment (**Fig 5D**). Upon release from the mitochondria, cytochrome c complexes with procaspase-9 and apoptotic protease activating factor 1, triggering the activation of a caspase cascade and eventually the dismantling of the cells. Indeed, BM-1197 treatment resulted in a strong cleavage of caspase-9 and -3 in cells (**Fig 5A**). Z-VAD-FMK, a pan-caspase inhibitor, diminished BM-1197-induced apoptosis, indicating that apoptosis induction by BM-1197 is caspase dependent (**Fig 5E**).

**Figure 6:**
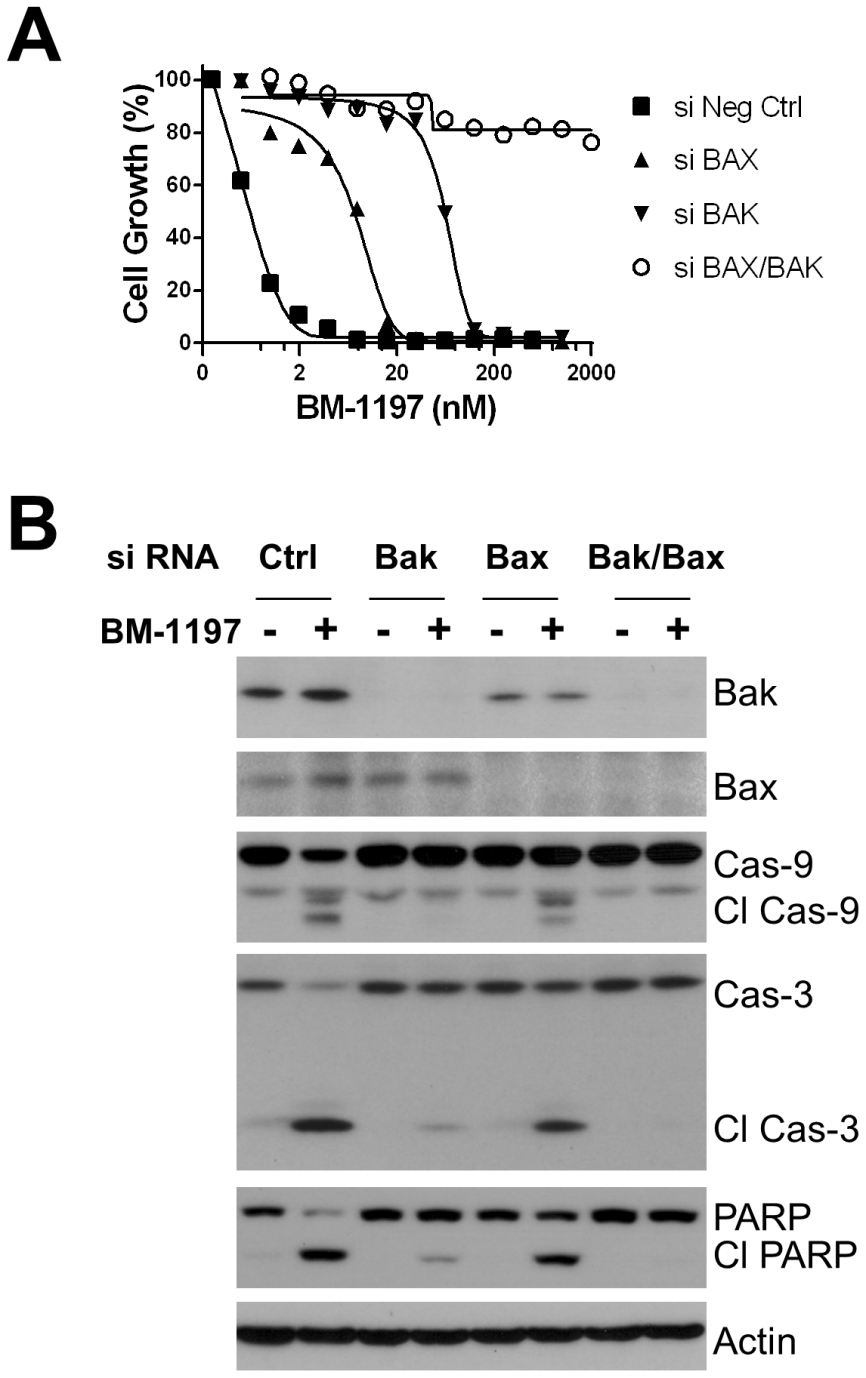
BM-1197 induces BAX/BAK-dependent apoptosis in MEF/*MCL1^−^* ^/*−*^ cells. (A) *MCL1^−/−^* cells were transfected with Bax- and/or Bak-specific siRNAs for 2 days then treated with BM-1197 for another 2 days. The growth-inhibitory activity of BM-1197 was evaluated by WST assay. Data are mean ± SD, and are representative of at least three independent experiments. (B) *MCL1^−/−^* cells were transfected with mouse Bax- and/or Bak-specific siRNAs for 2 days then treated with BM-1197 (50 nM) for 20 h. Whole cell extracts were analyzed by immunoblotting.

We investigated the role of Bax and Bak in BM-1197-induced apoptosis using gene-specific siRNAs targeting Bax or Bak. While depletion of Bak or Bax attenuates the BM-1197 activity in *MCL1^−/−^* MEF cells, simultaneous knocking down of Bax and Bak completely blocked BM-1197 activity in these cells (**Fig 6A–B**), indicating that BM-1197 induces apoptosis in a strictly Bax/Bak-dependent manner.

Collectively, these data demonstrate that BM-1197 is a *bona fide* Bcl-2/Bcl-xL inhibitor which induces mechanism-based apoptosis in MEF/*MCL1^−/−^* cells.

### BM-1197 exerts potent growth-inhibitory activity against small cell lung cancer (SCLC) cells *in vitro*

Previous studies have shown that ABT-737 and ABT-263 exert potent antitumor activities in SCLC cells [11], [13], [31] and thus we examined the activity of BM-1197 in a panel of 12 SCLC cell lines, with ABT-263 as a control. As shown in **Table 1**, in 12 SCLC cell lines tested, BM-1197 showed potent growth-inhibitory activities in 7 cell lines with IC_50_ values <100 nM (3–82 nM), moderate activity in 3 cell lines with IC_50_ values of ∼600 nM and weak activity in 2 cell lines with IC_50_ values >2000 nM. BM-1197 was 5-26-fold more potent than ABT-263 in the 4 cell lines most sensitive to BM-1197.

**Table 1:** Cell growth inhibitory activity of BM-1197 and ABT-263 against SCLC cell lines.

Cell Lines	BM-1197 (nM)	ABT-263 (nM)
H146	3±2	24±12
H1417	3±1	79±31
H1963	5±2	30±17
H211	6±5	66±50
H187	31±4	70±29
DMS79	54±30	430±343
H889	82±20	30±11
SW1271	306±116	>2000
DMS53	538±254	197±50
H82	698±354	>2000
DMS273	>2000	>2000
H196	>2000	>2000

The concentration of drugs that inhibits 50% of cell growth (IC_50_) compared to DMSO control after 96-h incubation with drugs was determined. The data represent means ± SD (n = 3–6).

**Figure 7:**
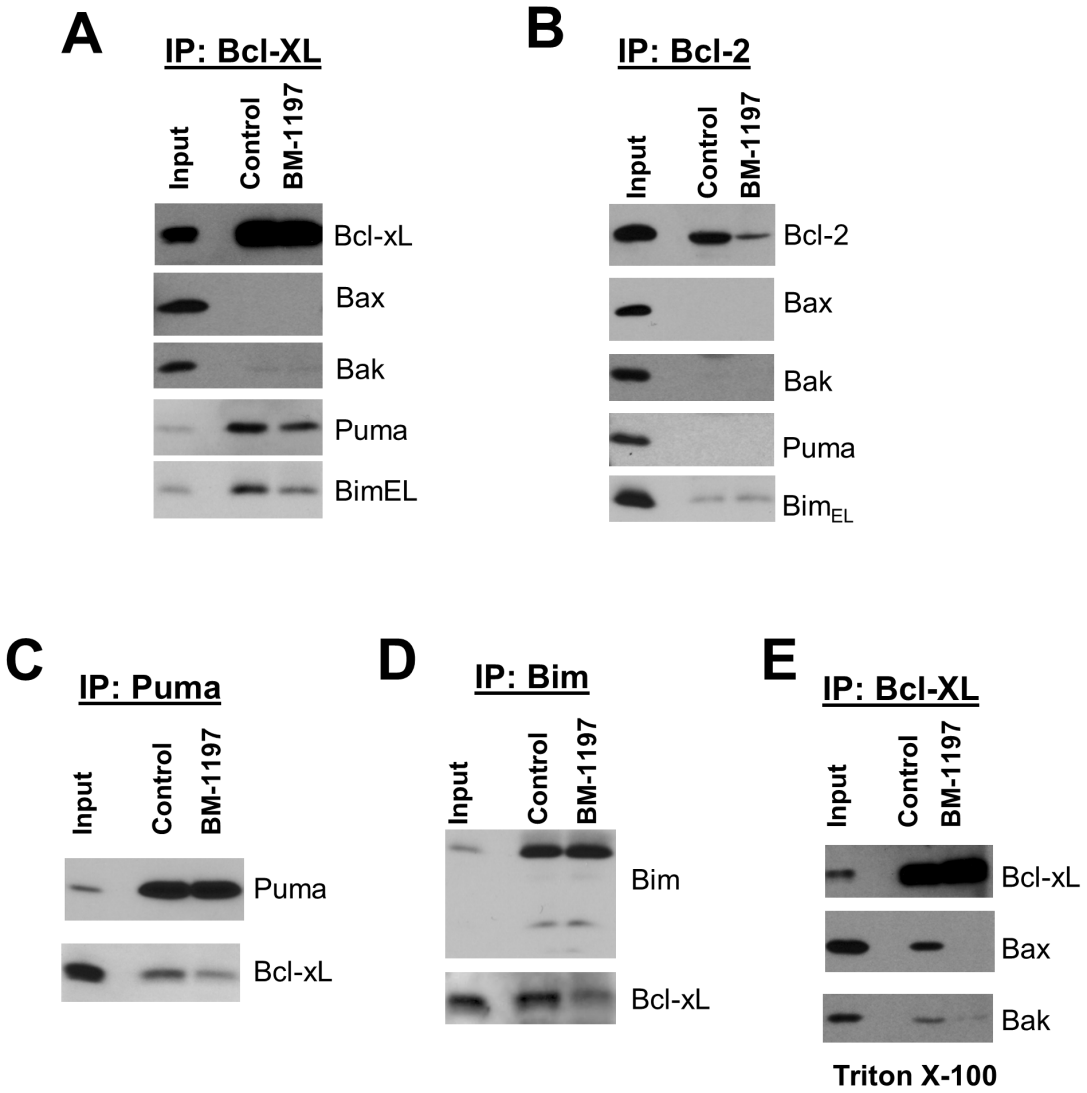
BM-1197 disrupts the association between BCL-XL and PUMA or BIM in H146 cells. (A–D) H146 cells were treated with DMSO (control) or BM-1197 (100 nM) for 2 h and CHAPS lysed whole cell extracts were immunoprecipitated with the indicated antibodies. The immunoprecipitates were analyzed by immunoblotting with the indicated antibodies. (E) H146 cells were treated with DMSO (control) or BM-1197 (100 nM) for 2 h and Triton X-100 lysed whole cell extracts were immunoprecipitated with anti-Bcl-xL antibody. The immunoprecipitates were analyzed by immunoblotting with the indicated antibodies.

To gain insight into the mechanism of action of BM-1197 in SCLC cell lines, the H146 cell line, which exhibits exquisite sensitivity to BM-1197 and ABT-263, was used for further analyses. Reciprocal immunoprecipitation assays were performed to determine the effect of BM-1197 on the heterodimeric interactions between anti- and pro-apoptotic Bcl-2 family proteins. It was found that BM-1197 attenuates the associations between Bcl-xL and BimEL or Puma (**Fig 7A–D**). Consistent with our observations in *MCL1^−/−^* MEF cells (**Fig 4A–C**), neither Bax nor Bak was found to be associated with Bcl-xL or Bcl-2 in untreated H146 cells when lysed with CHAPS (**Fig 7A–B**). However, when using Triton X-100 as the cell lysis detergent, Bax and Bak were found to be associated with Bcl-xL and BM-1197 efficiently displaced Bax and Bak from Bcl-xL protein (**Fig 7E**).

**Figure 8:**
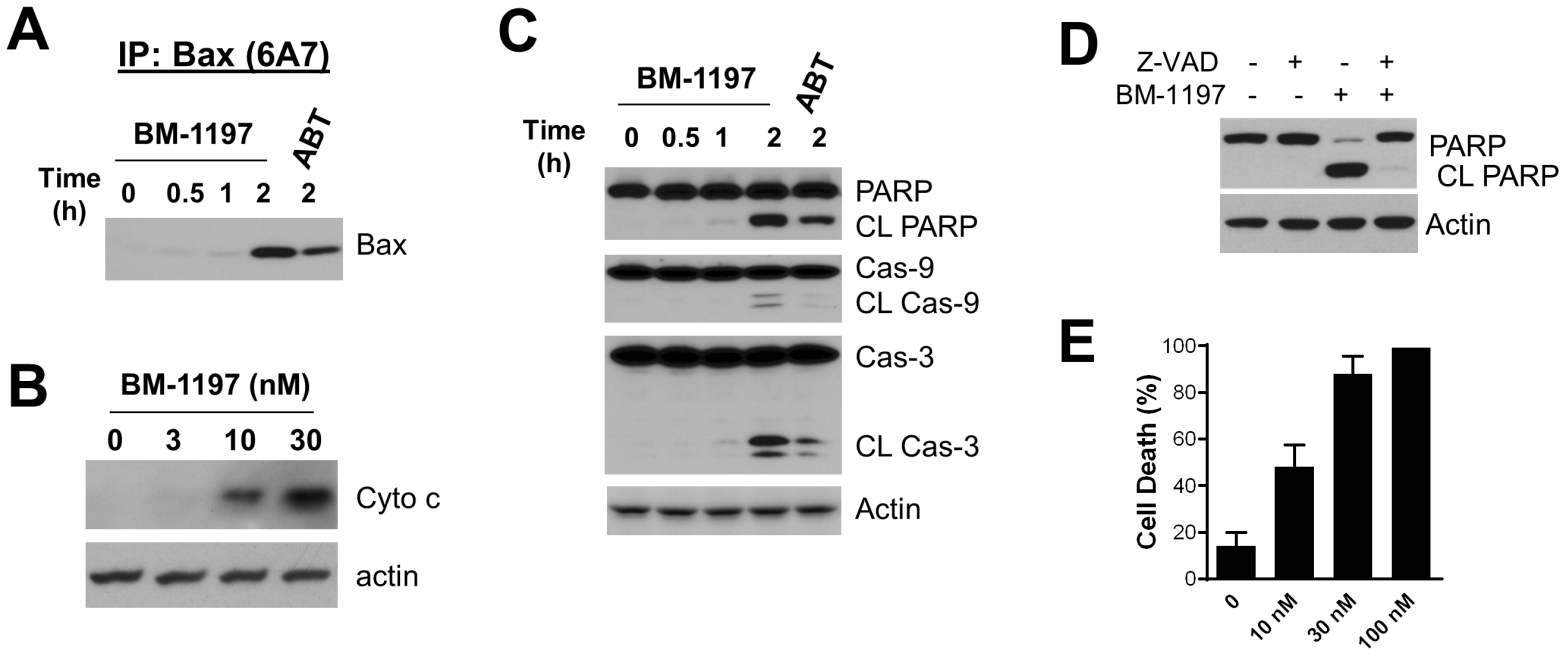
BM-1197 induces BAX translocation, cytochrome c release and cell death in H146 cells. (A) CHAPS lysed whole cell extracts from BM-1197 (100 nM)-treated H146 cells were immunoprecipitated with conformation-sensitive Bax specific antibody 6A7, and immunoprecipitates were analyzed by immunoblotting. (B) H146 cells were treated with BM-1197 for 2 h and cytosolic fraction was isolated for immunoblotting. (C) Cells were treated with BM-1197 (100 nM) or ABT-263 (100 nM) for the indicated time points and whole cell extracts were analyzed by immunoblotting. (D) H146 cells were pretreated with DMSO or 40 µM of Z-VAD for 2 h, then treated with 200 nM of BM-1197 for 4 h for immunoblotting analysis. (E) Cells were treated with BM-1197 for 1 day and cell death was evaluated by trypan blue exclusion assay. Data are expressed as mean ± SD (n = 3).

Immunoprecipitation with conformation-sensitive antibody against Bax showed that a rapid conformational change of Bax occurred upon treatment with BM-1197 (**Fig 8A**). Accordingly, immunoblotting demonstrated that BM-1197 potently induced cytochrome c release (**Fig 8B**), concomitant with the cleavage of caspase-9/-3 as well as PARP in H146 cells (**Fig 8C**). Similar to that observed in the MEF/*MCL1^−/−^* cells, the pan-caspase inhibitor Z-VAD-FMK blocked BM-1197-induced apoptosis (**Fig 8D**). Trypan blue staining demonstrated that BM-1197 induced H146 cell death in a dose-dependent manner (**Fig 8E**).

**Figure 9:**
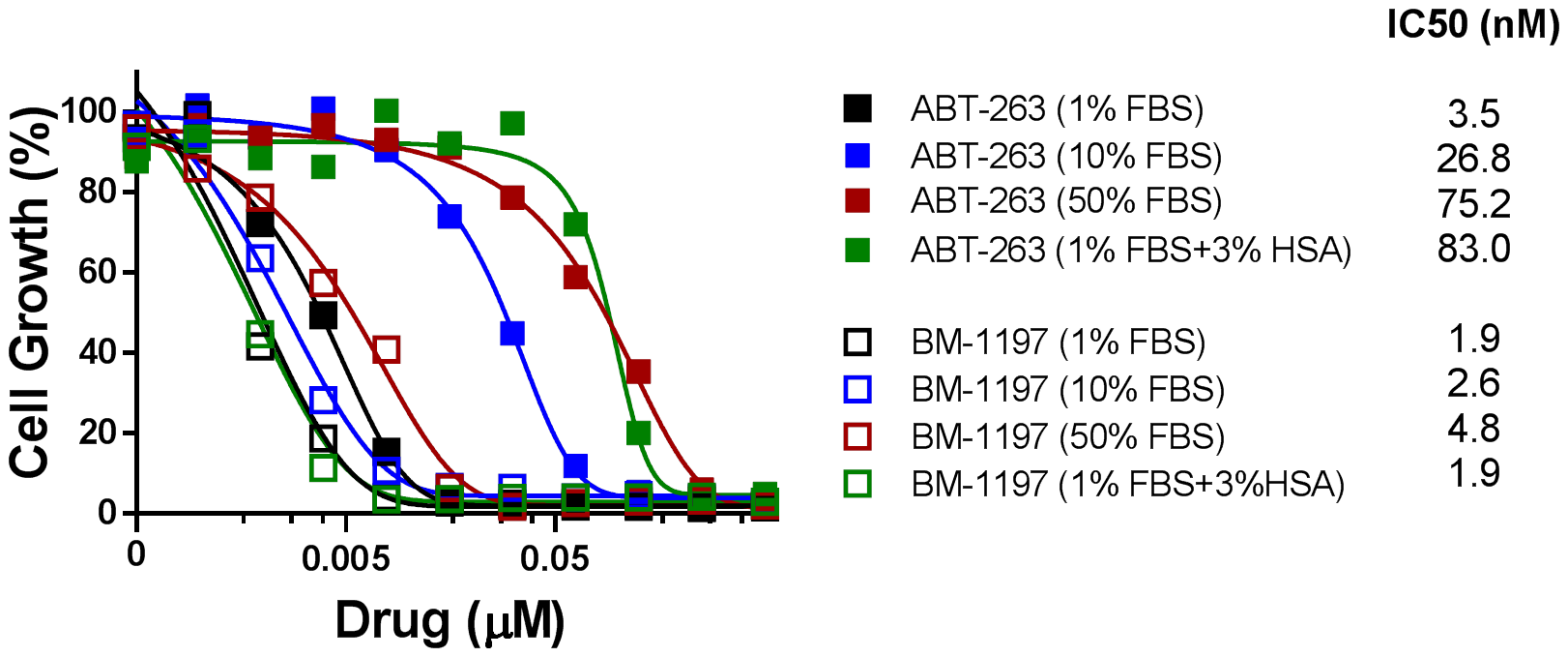
Albumin diminishes the growth-inhibitory activity of ABT-263 in H146 cells. H146 cells were treated with BM-1197 or ABT-263 in the presence of different concentrations of fetal bovine serum (FBS) or human serum albumin (HSA) for 4 days and the growth-inhibitory activities of BM-1197 and ABT-263 were evaluated by WST assay. Data are representative of two independent experiments.

The activity of ABT-263 was greatly reduced in whole blood compared to standard cell culture conditions due to its strong binding to albumin [22]. We thus evaluated the cell growth inhibitory activity of both BM-1197 and ABT-263 with different concentrations of fetal bovine serum (FBS) or human serum albumin (HSA) in cell culture medium. In agreement with the previous report [22], high concentrations of FBS or HSA in cell culture medium greatly reduced the growth-inhibitory activity of ABT-263 (**Fig 9**). However, FBS or HSA had a modest effect on the *in vitro* growth-inhibitory activity of BM-1197 (**Fig 9**), suggesting a relatively weak interaction between BM-1197 and albumin.

### Antitumor efficacy of BM-1197 in xenograft mouse models of SCLC cell lines

**Figure 10:**
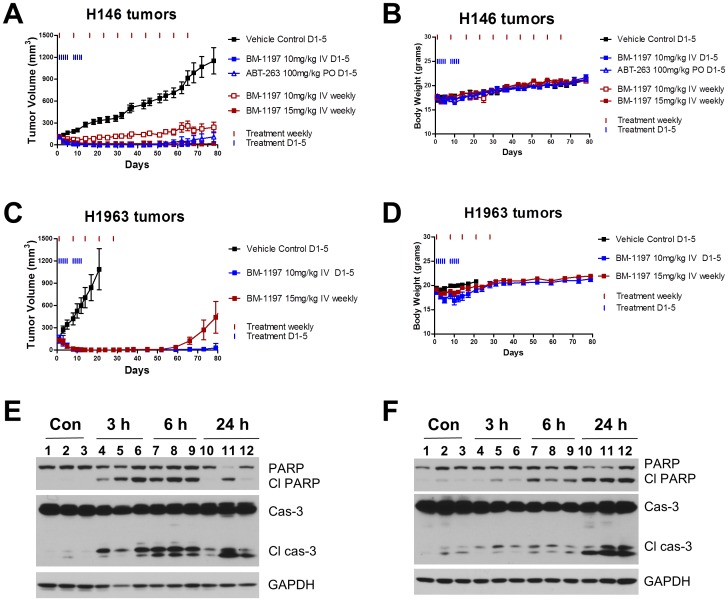
*In vivo* antitumor activity of BM-1197. (A–D) SCID mice bearing H146 or H1963 cells were treated with BM-1197 intravenously (IV) or ABT-263 orally (PO) at the indicated dosing schedules, and tumor volumes and body weights were measured every 2–3 days. Data shown are mean ± SEM for 6–8 mice. (E & F) SCID mice bearing H146 or H1963 cells were treated with 10 mg/kg of BM-1197 IV for the indicated times. Tumor lysates were analyzed by immunoblotting.

The antitumor efficacy of BM-1197 was evaluated in H146 and H1963, subcutaneous xenograft mouse models of SCLC cell lines. In the H146 tumor model, administration of BM-1197 IV daily at 10 mg/kg (5 days per week for 2 weeks) resulted in rapid and complete tumor regression in all 8 mice (**Fig 10A**). After two doses, mean tumor volume was decreased by 40% (from 114 mm^3^ to 69 mm^3^) and by 85% after 7 doses. All 8 mice remained tumor free for at least 32 days after the end of the treatment and 5 mice remained tumor free 60 days after the end of the treatment. Consistent with a previous report [13], ABT-263 at 100 mg/kg daily oral dosing (5 days per week) also regressed H146 tumors to a barely palpable state 14 days after the start of the treatment but the effect was less enduring than that observed for BM-1197. Four out of 8 of the tumors and 7 of the 8 tumors regrew 29 and 57 days after the last treatment with ABT-263, respectively (**Fig 10A**). In all these dose-schedules, animals experienced no statistically significant weight loss when compared to the animals in the control group and failed to show other signs of toxicity (**Fig 10B**).

We also evaluated the antitumor activity of BM-1197 with weekly, intravenous dosing in the H146 model. As shown in **Fig 10A**, a single 10 mg/kg dosing of BM-1197 reduced the mean tumor volume by 32% on day 7 but no further tumor regression was observed with additional dosing. In comparison, a single 15 mg/kg dosing of BM-1197 regressed H146 tumors by 66% on day 7. Complete tumor regression was observed in 4 of 8 mice after five weekly doses, and 7 of 8 mice after eight weekly doses (**Fig 10A**). Furthermore, 5 of 8 mice remained tumor free 50 days after the end of the weekly treatment.

In H1963 tumor model, BM-1197 at 10 mg/kg daily IV dosing (5 days per week) achieved complete tumor regression in all the 6 mice (**Fig 10C**). Seven days after the initiation of the treatment, the mean tumor volume was decreased by >90% and 10 days after the start of treatment, no mice had palpable tumors (**Fig 10C**). Although 1 of 6 mice had tumor regrown 40 days after the last dose, the other 5 mice remained tumor free for 61 days after the last dose. We also tested BM-1197 in H1963 xenograft model with weekly dosing. BM-1197 reduced the mean tumor volume by 66% after a single dose and achieved complete tumor regression after two doses (**Fig 10C**). The complete tumor regression lasted for 17 days in all mice and for 38 days in 5 out of 6 mice. As compared to the daily dosing at 10 mg/kg, the weekly dosing at 15 mg/kg was less effective in maintaining tumor-free status in mice. For example, on day 59, all mice in the group treated with weekly dosing had a re-occurring tumor but only one of six mice in the group treated with daily dosing had a palpable tumor (**Fig 10C**).

For both daily and weekly dose-schedules for BM-1197 in H1963 xenograft model, animals experienced <10% of weight loss and all animals gained weight after the treatment was completed. However, the weekly dose-schedule at 15 mg/kg caused less weight loss than the daily dosing schedule at 10 mg/kg (**Fig 10D**), suggesting a potential advantage for the weekly dosing schedule.

We analyzed biomarkers of apoptosis in H146 and H1963 tumors treated with a single dose of BM-1197. As shown in **Fig 10E and 10F**, BM-1197 at 10 mg/kg induced rapid and robust caspase-3 activation and PARP cleavage in H146 and H1963 tumors at time-point as early as 3 h with the effect persisting for at least 24 hr.

### On-target platelet toxicity of BM-1197 in mice

**Figure 11:**
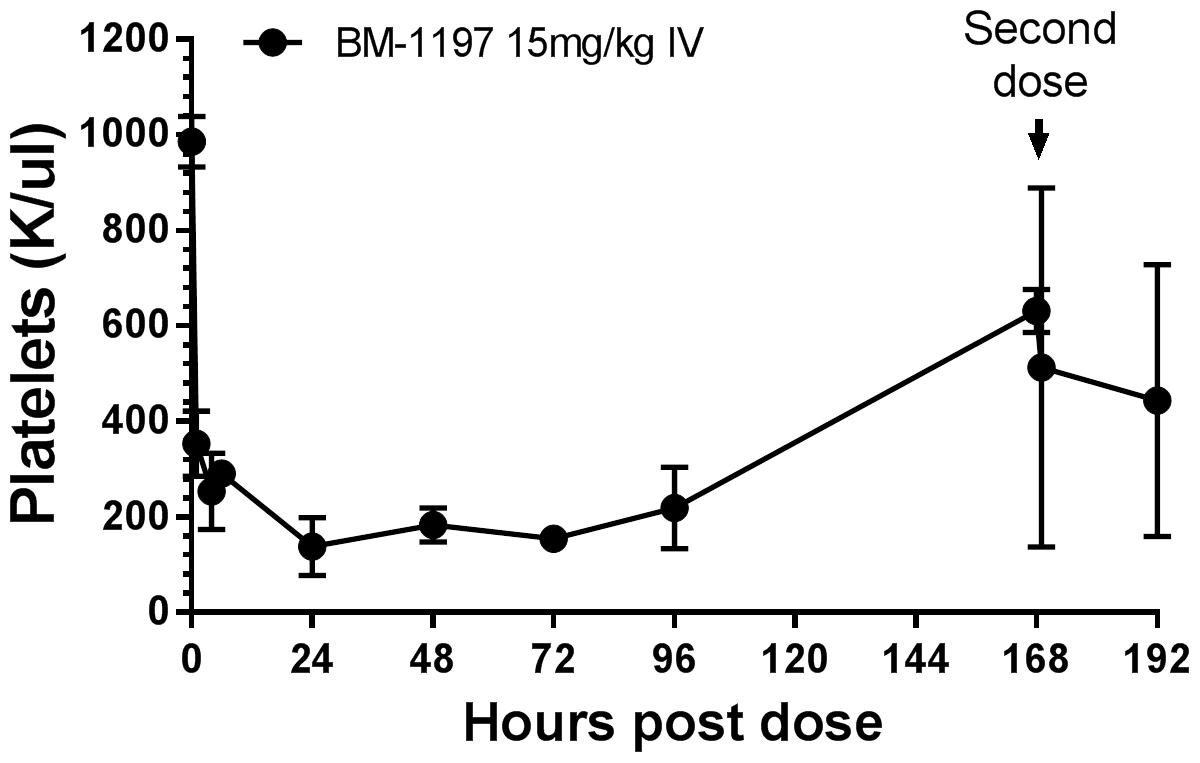
BM-1197 decreases platelet count in mice. BM-1197 was administered by IV bolus injected into the tail vein. At the indicated time points the mice were euthanized and blood was drawn for complete blood count. Data are mean ± SEM (n = 3).

Previous studies have shown that Bcl-xL is critical for survival of platelets and potent Bcl-xL inhibitors such as ABT-737, ABT-263, WEHI-539, and BXI-72 exert their on-target toxicity on the platelets (thrombocytopenia) [14], [19], [31], [32]. Consistent with its high affinity for BCL-xL, BM-1197 reduced the platelet count in mice; the lowest platelet count in all mice dosed at 15 mg/kg was 9.6×10^4^/µl at 24 h. The platelet count started to recover 24 h after dosing, consistent with the notion that aged platelets are more susceptible to apoptosis induction by inhibition of BCL-xL [32] (**Fig 11**). Despite the reduction of platelets by BM-1197, in our efficacy experiments we did not observe any bleeding in repeated daily or weekly intravenous doses with BM-1197 in mice. Taken together, our data suggest that BM-1197 indeed causes thrombocytopenia in mice but the effect is reversible even at highly efficacious doses.

### Depletion of *MCL1* sensitizes SCLC cells to BM-1197 induced cell death

**Figure 12:**
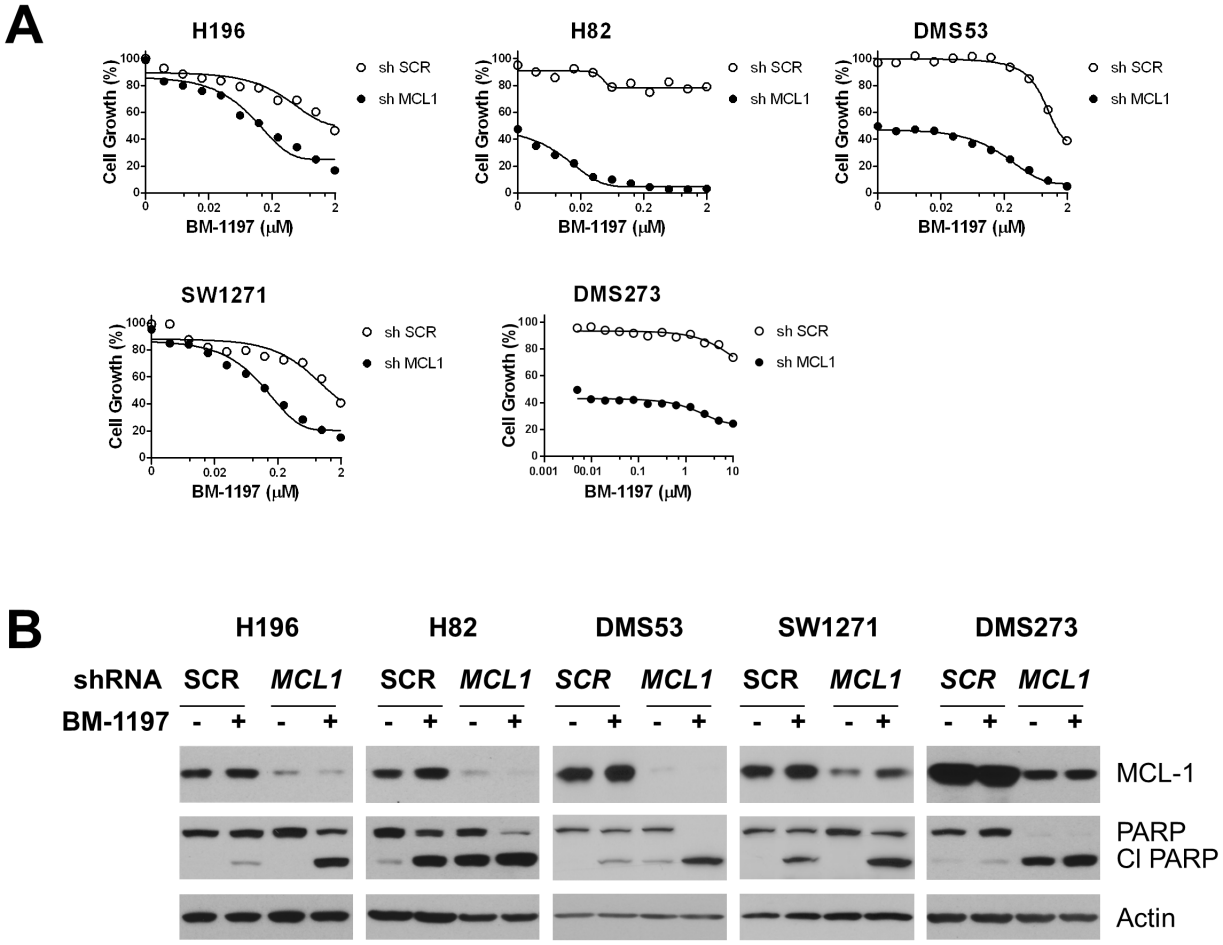
Effects of MCL1 knockdown on BM-1197-induced apoptosis. (A) Cells were transduced with lentiviral particles containing scrambled (SCR) or MCL1-specific shRNAs for 2 days, then treated with BM-1197 for 2 days. The growth-inhibitory activity of BM-1197 was assessed by WST assay. Data are representative of three independent experiments. (B) Cells were transduced with lentiviral particles containing scrambled (SCR) or MCL1-specific shRNAs for 2 days then treated with BM-1197 (100 nM) for 20 h before harvested for immunoblotting.

A number of studies have shown that Mcl-1 is a key resistant factor for ABT-737 and ABT-263 in a variety of tumor types [29], [30], [33], [34], [35]. Since BM-1197 has the same binding profile as ABT-263 and ABT-737, we evaluated modulation by Mcl-1 of BM-1197 responsiveness in SCLC cell lines. To this end, gene-specific shRNA of *MCL1* was used to knockdown its expression in several SCLC cell lines that are insensitive to BM-1197. Knocking down of *MCL1* had a variable effect on the growth of SCLC cells (**Fig 12A**). Notably, depletion of *MCL1* dramatically enhanced the inhibitory activity of BM-1197 in 4 of 5 BM-1197-insensitive cell lines (**Fig 12A**). Western blot analysis demonstrated that *MCL1* depletion significantly enhanced BM-1197-induced PARP cleavage in these cell lines (**Fig 12B**).

## Discussion

In this study, we investigated the mechanism of action and antitumor activity of BM-1197, a potent, selective, dual Bcl-2 and Bcl-xL inhibitor, *in vitro* and *in vivo*.

The study has provided several lines of evidence that BM-1197 is a potent, specific and *bona fide* Bcl-2/Bcl-xL inhibitor and exerts on-target activity: (1) it binds to Bcl-2 and Bcl-xL in biochemical assays with binding affinities at subnanomolar levels and shows a high specificity over Mcl-1; (2) it dissociates the heterodimeric interactions between pro-apoptotic and anti-apoptotic Bcl-2 family proteins in a cellular context; (3) it induces conformational change in the Bax protein; (4) it causes MOMP changes and cytochrome c release; (5) it induces caspase-9 cleavage and activation; and (6) it induces Bax/Bak-dependent apoptosis.

ABT-737 and ABT-263 have been shown to induce apoptosis in tumor cells by disrupting the interactions between anti- and pro-apoptotic Bcl-2 proteins [23], [36], [37], [38], [39]. Because BM-1197 induces apoptosis in a strictly Bak/Bax-dependent manner (**Fig. 6**), we first examined whether BM-1197 blocks the association of Bax/Bak with either Bcl-2 or Bcl-xL. Since the association between Bax and other Bcl-2 family members as well as the subcellular distribution of Bax can be altered by non-ionic detergents but not by zwitterionic detergents used to solubilize cells [27], [40], we employed the zwitterionic detergent CHAPS in our experiments. Our studies show that Bax or Bak does not co-immunoprecipitate with Bcl-2 or Bcl-xL protein when cells are lysed with CHAPS (**Fig 4A–C** **,** **7A–B**). Instead, Puma and Bim are associated with Bcl-xL in *MCL1*-deficient MEF cells and/or H146 cells. Significantly, the association between Bcl-xL and Puma or Bim is reduced by BM-1197 (**Fig 4D-E** **,** **7A–D**). Since BH3-only proteins Bim and Puma can directly bind to and activate Bak and Bax, our data suggest that BM-1197 induces Bax/Bak-dependent apoptosis through release of Puma and Bim from its association with Bcl-xL (or with Bcl-2), and subsequent activation of Bax and Bak by Puma and/or Bim.

It is well accepted that the mitochondrial outer membrane plays an active role in modulating the conformation, interactions, and functions of the Bcl-2 family proteins, and apoptotic stimuli can also lead to dynamic conformational changes in some Bcl-2 family proteins. Induction of apoptosis shifts the subcellular locations of Bax and Bcl-xL from soluble to membrane-bound forms [41]. It has been reported that the cytosolic Bax is not associated with Bcl-2 or Bcl-xL and induction of apoptosis fails to result in appreciable interaction between Bax and Bcl-2 or Bcl-xL [40]. Intriguingly, when using Triton X-100 as the cell lysis detergent, Bax and/or Bak were found to be associated with Bcl-xL in MEF *MCL1* ^−/−^ and H146 cells and BM-1197 efficiently displaced Bax and/or Bak from Bcl-xL protein (**Fig 4B** **,** **7E**). Therefore, different experimental approaches could lead to different models of induction of apoptosis by Bcl-2/Bcl-xL inhibitors, and care must be taken in elucidation of the interactions between the Bcl-2 family proteins in conditions where membranes are destroyed [42].

Our data show that BM-1197 exerts potent cell growth inhibitory activity against 7 out of 12 SCLC cell lines *in vitro*, with IC_50_ values <100 nM (3–82 nM). Among the 4 most sensitive cell lines, BM-1197 is 5–26 times more potent than ABT-263, a gold standard for Bcl-2/Bcl-xL inhibitors. *In vivo*, BM-1197 achieves rapid and complete tumor regression in 100% of mice treated with 10 mg/kg, daily dosing for 2 weeks in both H146 and H1963 xenograft models. The complete tumor regression is long-lasting in both models. In fact, 5 of 8 mice bearing H146 xenograft tumors achieved a “therapeutic cure” and remained tumor-free 60 days after the end of the treatment. In both models, BM-1197 was well tolerated, the mice experienced <10% weight loss during the entire experiment and gained weight after the treatment was completed. In comparison, ABT-263 at 100 mg/kg, daily oral dosing also achieved complete tumor regression in H146 xenograft model but the effect was less persistent than with BM-1197 at 10 mg/kg, daily intravenous dosing.

For drugs that need to be administered intravenously, daily dosing may be difficult in the clinic, and consequently we examined a weekly dosing-schedule for BM-1197 in both H146 and H1963 models. BM-1197, with 15 mg/kg weekly dosing achieved complete tumor regression in 7 out of 8 mice in a H146 xenograft model and in 100% of mice in H1963 xenograft model. Additionally, mice had less weight loss in the weekly dosing schedule at 15 mg/kg of BM-1197 than in the daily dosing schedule at 10 mg/kg of BM-1197. Our data thus suggest that less frequent (weekly) dosing may be used in the clinic for highly potent Bcl-2/Bcl-xL inhibitors to achieve strong antitumor activity, which may not only make the treatment more feasible for patients but also reduce the potential side effects.

In preclinical models, ABT-263 has demonstrated potent antitumor activity and clinical efficacy in Bcl-2-driven tumors, such as chronic lymphocytic leukemia [11], [13], [21], [43]. Although ABT-737 and ABT-263 have also demonstrated promising antitumor activity in cell line-based preclinical models of SCLC [11], [13], [43], they are much less effective in primary patient-derived xenograft (PDX) models of SCLC [44]. Furthermore, ABT-263 showed low single-agent clinical activity in a Phase II clinical trial of SCLC patients [45], [46]. Therefore, the preclinical data derived from SCLC PDX models and clinical data for ABT-263 suggest that a potent Bcl-2/Bcl-xL dual specific inhibitor may have very limited clinical utility as a single agent for the treatment of SCLC patients. However, there are a number of possible reasons to account for the minimal activity for ABT-263 in SCLC PDX models and in SCLC patients. First, ABT-263 has been shown to be less effective in antagonizing Bcl-xL than BCL-2 in cell-based assays, which agrees with the fact that ABT-263 is primarily effective against Bcl-2 driven tumors in the clinic. Second, those three SCLC PDX models used to evaluate ABT-737/ABT-263 have low expression of Bcl-2, suggesting that they are not Bcl-2 driven tumors. Third, the status of expression of Mcl-1 in those three SCLC PDX models used to evaluate ABT-263 is not known and these models may be intrinsically resistant to potent and specific Bcl-2/Bcl-xL inhibitors if they have high expression of Mcl-1. Hence, it is possible that a more potent and efficacious Bcl-2/Bcl-xL dual inhibitor may still have a promising therapeutic potential against SCLC tumors with a high expression of Bcl-2 and/or Bcl-xL and a low expression of Mcl-1 as a single agent. Our *in vitro* cell growth data showed that BM-1197 is about 8-times more effective than ABT-263 in MEF cells with Mcl-1 deletion and is 5-26-fold more potent than ABT-263 in 4 most sensitive SCLC cancer cell lines. Our *in vivo* data showed that BM-1197 with either daily or weekly dosing is capable of achieving complete tumor regression in two SCLC xenograft models. Furthermore, BM-1197 at 10 mg/kg daily intravenously dosing for 2 weeks is more effective than ABT-263 at 100 mg/kg daily oral dosing for 2 weeks in achieving long-term tumor regression in the H146 xenograft model. Our *in vitro* and *in vivo* preclinical data strongly suggest the potential value of BM-1197 as a single agent for the treatment of SCLC, despite the minimal clinical activity of ABT-263 in SCLC patients.

One major challenge for the development of dual Bcl-2/Bcl-xL inhibitors is the on-target cytotoxicity on platelets, which rely on Bcl-xL for survival [32], [47]. Consistent with its subnanomolar binding affinity to Bcl-xL, BM-1197 at 15 mg/kg effectively decreases platelet count in mice (**Fig 11**). However, this effect was reversible in all the mice. These data, coupled with the fact that BM-1197 can achieve complete tumor regression with weekly dosing in two different xenograft models, suggest that BM-1197 may possess an acceptable toxicity profile for cancer treatment, especially with infrequent (e.g. weekly) dosing schedules.

Although BM-1197 is very effective (IC_50_ <100 nM) in inhibition of cell growth in 7 out of 12 SCLC cell lines evaluated, it is modestly effective or ineffective against 5 other SCLC cell lines. Therefore, for successful development of BM-1197, it is critical to identify the key resistance factor(s) and to develop rational combination strategies. Since BM-1197 selectively targets Bcl-2 and Bcl-xL and does not target Mcl-1, we have investigated if Mcl-1 mediates the resistance of BM-1197 in SCLC cell lines. Indeed, efficient knock-down of *MCL1* by lentiviral shRNAs greatly enhances the inhibitory activity of BM-1197 in 4 of 5 resistant SCLC cell lines (**Fig 12A**) and the ability of BM-1197 to induce PARP cleavage (apoptosis) in these 4 cell lines (**Fig 12B**). These data suggest that even when BM-1197 is ineffective as a single agent, its combination with an agent that can target Mcl-1 directly or indirectly can be an effective therapy for the treatment of SCLC or other types of human cancer.

In summary, our preclinical studies demonstrate that BM-1197 is a highly potent, efficacious and *bona fide* Bcl-2/Bcl-xL inhibitor warranting further investigation as a new anticancer drug.
